# Normal inflammatory markers and acute appendicitis: a national multicentre prospective cohort analysis

**DOI:** 10.1007/s00384-021-03933-7

**Published:** 2021-04-27

**Authors:** J. de Jonge, J. C. G. Scheijmans, C. C. van Rossem, A. A. W. van Geloven, M. A. Boermeester, W. A. Bemelman, on behalf of the Snapshot Appendicitis Collaborative Study group, G. J. Van Acker, B. Akkermans, G. J. Akkersdijk, G. D. Algie, J. H. Allema, C. S. Andeweg, N. Appeldoorn, J. G. van Baal, C. M. den Bakker, S. A. Bartels, C. van den Berg, B. Boekestijn, F. C. den Boer, D. Boerma, A. L. van den Boom, M. C. Boute, S. A. Bouwense, J. Bransen, F. A. van Brussel, O. R. Busch, S. M. de Castro, H. A. Cense, C. Croese, T. van Dalen, I. Dawson, E. van Dessel, R. Dettmers, N. Dhar, F. Y. Dohmen, K. W. van Dongen, P. van Duijvendijk, R. R. Dulfer, B. J. Dwars, J. P. Eerenberg, M. van der Elst, E. van den Ende, L. M. Fassaert, J. T. Fikkers, J. W. Foppen, E. J. Furnee, F. P. Garssen, M. F. Gerhards, H. van Goor, J. S. de Graaf, L. J. Graat, J. Groot, A. C. van der Ham, J. F. Hamming, J. T. Hamminga, E. van der Harst, J. Heemskerk, A. Heijne, J. T. Heikens, E. Heineman, R. Hertogs, E. van Heurn, L. C. van den Hil, A. G. Hooftwijk, C. C. Hulsker, D. R. Hunen, M. S. Ibelings, J. M. Klaase, R. Klicks, L. Knaapen, R. T. Kortekaas, F. Kruyt, S. Kwant, S. S. Lases, T. Lettinga, A. Loupatty, R. A. Matthijsen, R. C. Minnee, B. Mirck, L. Mitalas, D. Moes, A. M. Moorman, V. B. Nieuwenhuijs, G. A. P. Nieuwenhuijzen, P. D. Nijk, J. M. Omloo, A. G. Ottenhof, H. W. Palamba, D. L. van der Peet, I. T. Pereboom, P. W. Plaisier, A. P. van der Ploeg, M. H. Raber, M. M. Reijen, H. Rijna, C. Rosman, R. M. Roumen, R. F. Scmitz, A. P. Schouten van der Velden, W. H. Scheurs, T. A. Sigterman, H. J. Smeets, D. J. Sonnevled, M. N. Sosef, S. F. Spoor, L. P. Stassen, L. van Steensel, E. Stortelder, J. Straatman, H. J. van Susante, D. E. Suykerbuyk de Hoog, C. Terwisscha van Scheltinga, B. R. Toorenvliet, P. C. Verbeek, M. Verseveld, J. H. Volders, M. R. Vriens, P. W. Vriens, B. C. Vrouenraets, B. J. van de Wall, J. A. Wegdam, E. Westerduin, J. J. Wever, N. A. Wijfels, B. P. Wijnhoven, T. A. Winkel, A. M. van der Zee D. C. Zeillemaker, C. Zietse

**Affiliations:** 1 Department of Surgery, Tergooi Hospital Hilversum, Hilversum, The Netherlands; 2 Department of Surgery, Amsterdam UMC, location AMC, Amsterdam Gastroenterology Endocrinology Metabolism, University of Amsterdam, Amsterdam, the Netherlands; 3 Department of surgery, Maasstad Hospital Rotterdam, Rotterdam, The Netherlands; 4 Department of surgery, Amsterdam University Medical Centers, location Amsterdam Medical Center, Amsterdam, the Netherlands

**Keywords:** Appendicitis, Inflammatory markers, Complicated appendicitis, C-reactive protein

## Abstract

**Purpose:**

For the diagnosis of acute appendicitis, the combination of clinical and laboratory variables achieves high diagnostic accuracy. Nevertheless, appendicitis can present with normal laboratory tests of inflammation. The aim of this study was to investigate the incidence of normal inflammatory markers in patients operated for acute appendicitis.

**Methods:**

This is an analysis of data from a prospective, multicentre SNAPSHOT cohort study of patients with suspected acute appendicitis. Only patients with histopathologically proven acute appendicitis were included. Adult patients with acute appendicitis and normal preoperative inflammatory markers were explored further in terms of abdominal complaints, preoperative imaging results and intraoperative assessment of the degree of inflammation and compared to those with elevated inflammatory markers.

**Results:**

Between June and July 2014, 1303 adult patients with histopathologically proven acute appendicitis were included. In only 23 of 1303 patients (1.8%) with proven appendicitis, both preoperative white blood cell count and C-reactive protein levels were normal. Migration of pain was reported less frequently in patients with normal inflammatory markers compared to those with elevated inflammatory marker levels (17.4% *versus* 43.0%, *p* = 0.01). Characteristics like fever, duration of symptoms and localized peritonitis were comparable. Only 4 patients with normal inflammatory markers (0.3% overall) had complicated appendicitis at histopathological evaluation.

**Conclusion:**

Combined normal WBC and CRP levels are seen in about 2 per 100 patients with confirmed acute appendicitis and can, although rarely, be found in patients with complicated appendicitis.

**Supplementary Information:**

The online version contains supplementary material available at 10.1007/s00384-021-03933-7.

## Introduction

Acute appendicitis is often suspected in patients presenting with acute abdominal pain in the right lower quadrant. White blood cell count (WBC) and c-reactive protein (CRP) levels are commonly used to make the diagnosis more or less likely. Clinical and laboratory variables, although weak discriminators individually, achieve high diagnostic accuracy when combined [1–3]. Still, ruling in acute appendicitis or completely ruling out acute appendicitis based on merely the inflammatory markers remains difficult [4]. Therefore, when suspicion is raised after physical examination and laboratory tests, imaging by means of ultrasonography (US), computed tomography (CT) or magnetic resonance imaging (MRI) could be performed. Routine imaging in patients suspected for acute appendicitis leads to a higher diagnostic accuracy and a significantly lower risk for a normal appendix at appendectomy [5, 6].

In addition, imaging appears important in differentiating between uncomplicated (phlegmonous) and complicated (perforated or gangrenous) appendicitis. Combined clinical and imaging features are used in predicting scores for this differentiation [4]. Differentiating is relevant, because more and more studies substantiate the difference in pathophysiology between uncomplicated and complicated appendicitis, leading to different recommendations for management of appendicitis. Former trials suggest that uncomplicated appendicitis could probably be treated by antibiotics only [7–11], while new national guidelines advice surgery [12, 13] within 8 h in patients suspected for complicated appendicitis based on a recent systematic review [14]. Since imaging plays an important role in differentiating between these two types and consequently in the treatment options, imaging should be done routinely in patients clinically suspected for appendicitis. Nevertheless, due to costs, overcrowding and radiation exposure, not all clinically suspected patients undergo imaging. Several studies suggest that normal levels of WBC and CRP rule out appendicitis [15, 16]. An expectative approach, with possible reassessment the day after initial presentation, is therefore often chosen. On the contrary, other studies state that normal levels of biochemical tests cannot rule out appendicitis completely [4, 17].

The aim of this study is to describe the proportion of patients with normal preoperative inflammatory markers among patients with histopathologically proven acute appendicitis.

## Methods

### Study design, setting and participants

**Fig. 1:**
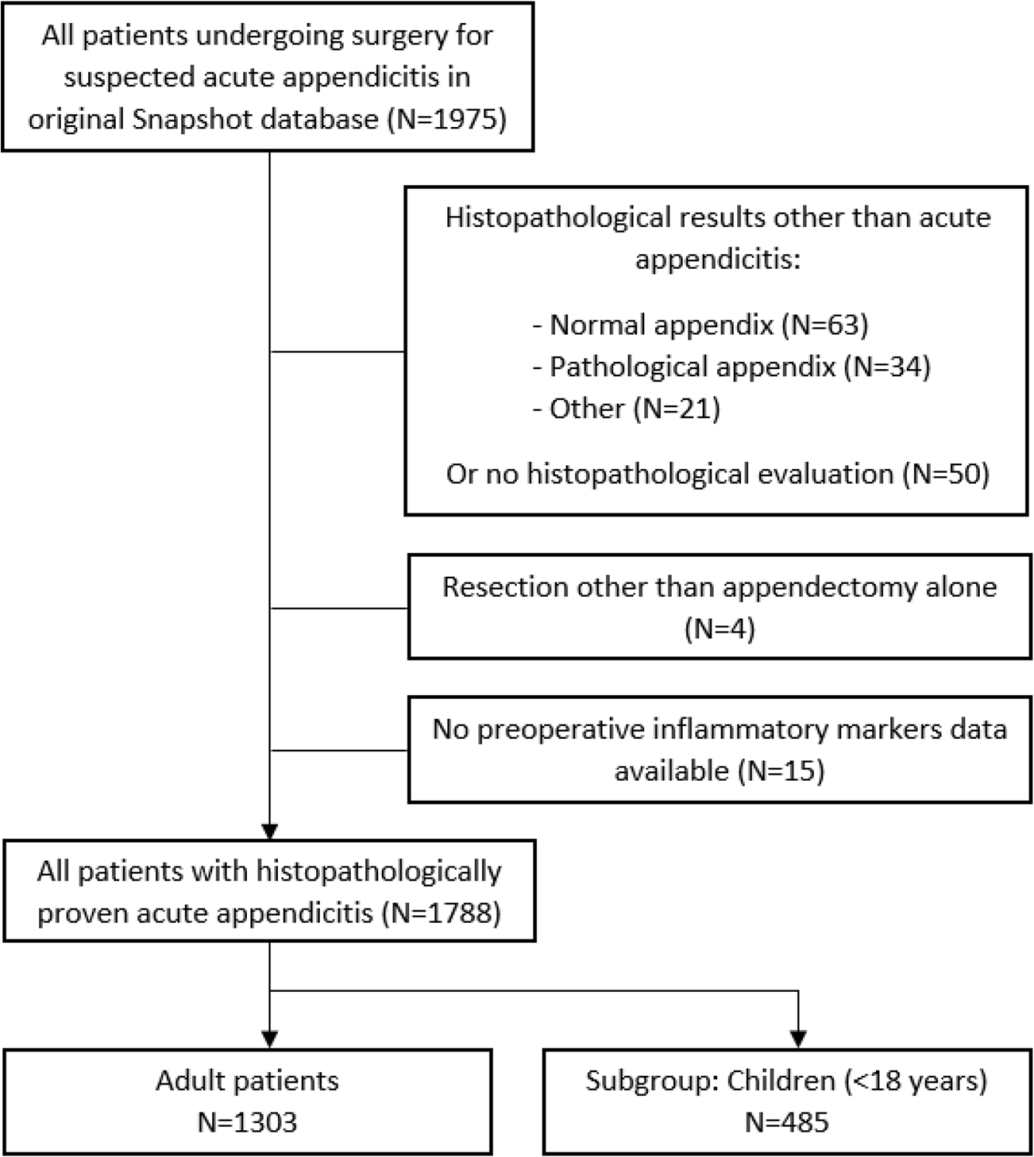
Flowchart: patients eligible for present cohort study

Data was used from the SNAPSHOT appendicitis study [6]. This was a prospective, nationwide audit that included all patients undergoing surgery for suspected acute appendicitis, conducted in a 2-month period (June and July 2014) in 62 Dutch academic and general community hospitals. The purpose of this audit was to describe the variation in practice and outcomes of acute appendicitis care in the Netherlands [6]. Data were collected about diagnostic tests, treatment and postoperative outcomes. The medical ethics committee in the Academic Medical Center in Amsterdam approved the study protocol. Owing to the observational, non-interventional study design, informed consent requirement was not necessary. Both adults and children were included in the SNAPSHOT cohort. For current analysis, only patients with histopathological proven acute appendicitis after appendectomy were included. The primary analysis focused on adult patients (≥ 18 years). Paediatric patients were examined as a separate group. Also gender as variable in young adults (< 40 years) was explored in subgroup analysis. Patients who underwent surgery other than appendectomy and those missing inflammatory parameters were excluded (Fig. 1).

### Data extraction

Baseline patient characteristics (i.e. age, gender, ASA-classification (I–II or III–IV)) were prospectively registered as well as several pre-, intra- and postoperative variables: pre-hospital symptom time (< or > 2 days), migration of pain, nausea, vomiting, fever (temperature < or > 38.5°), extent of peritonitis (local, diffuse), white blood cell count (WBC – positive if > 11 × 10^9^/L) and c-reactive protein (CRP – positive if ≥ 5 mg/L) at presentation, time between presentation and operation (hours), surgical approach (laparoscopic or open), duration of surgery (min), results of radiological imaging (inconclusive, uncomplicated, gangrenous or perforated appendicitis, inflammatory mass +/− abscess, no imaging performed), length of hospital stay (LOS), intraoperative findings (uncomplicated-, gangrenous- or perforated appendicitis, tumour of the appendix or iatrogenic perforation of the appendix) and histopathological findings.

### Outcome parameters and definitions

The main outcomes in this study were the number and proportion of patients with histopathological proven acute appendicitis after appendectomy and normal preoperative inflammatory markers. Inflammatory markers were considered to be normal if WBC was ≤ 11 × 10^9^/L and CRP < 5 mg/L. These cut-off values are based on hospital guidelines. Secondary outcomes were preoperative imaging results, the intraoperative degree of inflammation of the appendix (uncomplicated-, gangrenous- or perforated appendicitis) and the histopathological findings. Uncomplicated appendicitis is defined as non-gangrenous and non-perforated appendicitis. Gangrenous appendicitis was defined as gangrene or necrosis of the appendix. Both gangrene and perforations were scored by pathologist and surgeon. In case of discrepancy, necrosis had to be confirmed by the pathologist for the final diagnosis of gangrenous appendicitis, while surgical confirmation of a perforation was required for the final diagnosis of perforated appendicitis, as it was considered a surgical diagnosis. No standard report was used for histological assessment; pathologists recorded their findings according to local protocols.

### Statistical analysis

In univariable analysis, outcomes were compared between patients without and with elevated inflammatory markers with the Chi-square or Fisher’s exact test in case of categorical variables and the independent samples Student’s *t*-test or Mann-Whitney test in case of continuous variables, as appropriate. A 2-sided*p* < 0.05 was considered significant. All data analyses were performed in SPSS version 21 (IBM, Armonk, New York, USA). This manuscript was written using the Strengthening the Reporting of OBservational studies in Epidemiology (STROBE) statement checklist [18].

## Results

Between June and July 2014, 1975 patients who underwent surgery for suspected acute appendicitis were prospectively included in our database. Of these patients, 1807 had histopathological proven appendicitis postoperatively (Fig. 1). In 4 patients, acute appendicitis was seen, but a resection other than appendectomy was performed (2 ileocecal resections and 2 right hemicolectomies). In 15 of the remaining 1803 patients, the results of either WBC or CRP were missing. A subgroup of 485 children (< 18 years) was excluded for primary analysis, leaving 1303 adult patients with histopathological proven appendicitis. Of these patients, the median age was 39 years (IQR 27 to 54) and 652 patients (50.0%) were male. All baseline characteristics of the cohort are described in Table 1.

**Table 1:** Baseline characteristics of patients with and without elevated inflammatory markers

Variable		Total (*n* = 1303)	Patients without elevated inflammatory markers (*n* = 23)	Patients with elevated inflammatory markers (*n* = 1280)	*p* Value
Age, median (IQR) in years *Missing n = 0*		39 (27-54)	31 (27-51)	39 (27-54)	0.31
Sex, male (%) *Missing n = 0*		652 (50.0)	9 (39.1)	643 (50.2)	0.29
ASA (%) *Missing n = 0*	I–II	1254 (96.2)	22 (95.7)	1332 (96.3	0.59*
	III–IV	49 (3.8)	1 (4.3)	48 (3.8)	
Duration of symptoms < 2 or > 2 days (%) *Missing n = 30*	< 2 days	1032 (79.2)	16 (69.6)	1016 (79.4)	0.18*
	> 2 days	241 (18.5)	7 (30.4)	234 (18.3)	
Migration of pain (%) *Missing n = 38*		555 (42.6)	4 (17.4)	551 (43.0)	0.01*
Nausea (%) *Missing n = 31*		888 (68.2)	15 (65.2)	873 (68.2)	0.63
Vomiting (%) *Missing n = 27*		448 (34.4)	5 (21.7)	443 (34.6)	0.18
Fever, ≥ 38.5 ° Celsius (%) *Missing n = 72*		238 (18.3)	1 (4.3)	237 (18.5)	0.10*
Peritonitis (%) *Missing n = 48*	Localized	1129 (86.6)	18 (78.3)	1111 (86.8)	0.46*
	Diffuse	126 (9.7)	3 (13.0)	123 (9.6)	
Inflammatory markers *Missing n = 0*	WBC, mean (SD) 10˄9/L	13.9 (4.5)	8.5 (1.8)	14.0 (4.5)	<0.001
	CRP, median (IQR) mg/L	39 (13-86)	2 (1-4)	40 (15-87)	<0.001
Time to surgery, median (IQR) in hours *Missing n = 22*		7.1 (4.5-13.6)	7.1 (3.3-17.8)	7.1 (4.5-13.5)	0.82
Type of appendectomy ITT (%) *Missing n = 0*	Laparoscopic	1029 (79.0)	15 (65.2)	1014 (19.2)	0.12*
	Open	268 (20.6)	8 (34.8)	160 (20.3)	
Duration of surgery, median (IQR) in min *Missing n = 55*		42 (32–56)	34 (28–47)	43 (32–56)	0.08
LOS, median (IQR) in days *Missing n = 0*		2 (1–4)	2 (1–3)	2 (1–4)	0.90

Abbreviations: *ASA* American Society of Anesthesiologists; *IQR* interquartile range, *ITT* intention to treat, *LOS* length of stay

*Fisher’s exact test was used

### Pre-operative inflammatory markers

The median WBC was 13.9 10^9^/L (SD 4.5) at presentation at the emergency department. In 347 patients (26.6%), the WBC was normal (< 11.0 × 10^9^/L). The median CRP level of all patients was 39 (IQR 13 to 86) mg/L. In 140 of 1303 patients (10.7%), the CRP level was normal (< 5 mg/L). In only 23 of 1303 patients (1.8%), both WBC and CRP levels were normal. Migration of pain was reported less frequently in these patients compared to those with elevated inflammatory markers (17.4% *versus* 43.0%, *p* = 0.010). Other baseline characteristics, e.g. duration of symptoms or fever, were comparable (Table 1).

### Pre-operative imaging results

Only 2 of 1303 patients (0.2%) were operated without imaging. None of these patients had normal WBC and CRP levels (Table 2a). All 23 patients with normal inflammatory markers underwent imaging because of suspected acute appendicitis based on clinical symptoms. Of these patients, 21 patients underwent ultrasound, in seven cases followed by CT and in one followed by MRI, all because of inconclusive US results, and 2 patients underwent only CT. After imaging, 20 patients were suspected of having uncomplicated appendicitis, one of perforated appendicitis and 2 of an inflammatory mass.

**Table 2:** Imaging results and intraoperative and histopathological findings

Variable	Total (*n* = 1303)	Patients without elevated inflammatory markers (*n* = 23)	Patients with elevated inflammatory markers (*n* = 1280)
a. Imaging results			
Inconclusive (%)	49 (3.8)	-	49 (3.8)
Uncomplicated appendicitis (%)	1041 (79.9)	20 (87.0)	1021 (79.8)
Perforated appendicitis (%)	139 (10.7)	1 (4.3)	138 (10.8)
Inflammatory mass (%)*	64 (4.9)	2 (8.7)	62 (4.8)
Inflammatory mass with abscess (%)**	8 (0.6)	-	8 (0.6)
No imaging (%)	2 (0.2)	-	2 (0.2)
b. Intraoperative findings			
Normal appendix (%)	6 (0.5)	-	6 (0.5)
Uncomplicated appendicitis (%)	873 (67.0)	19 (82.6)	854 (66.7)
Gangrenous appendicitis (%)	136 (10.4)	3 (13.0)	133 (10.4)
Perforated appendicitis (%)	260 (20.0)	1 (4.3)	259 (20.2)
Iatrogenic perforation (%)	28 (2.1)	-	28 (2.2)
c. Histopathological findings			
Uncomplicated appendicitis (%)	1015 (77.9)	19 (82.6)	996 (77.8)
Gangrenous appendicitis (%)	137 (10.5)	2 (8.7)	135 (10.5)
Perforated appendicitis (%)	151 (11.6)	2 (8.7)	149 (11.6)

*Intraoperatively, 27 of these patients had perforated or gangrenous appendicitis, of which 18 had purulent peritonitis; 37 patients were diagnosed as uncomplicated appendicitis, but in 7 of these cases purulent peritonitis was seen

**Intraoperatively, 4 of these patients had a perforated appendix, one a gangrenous appendix and three had uncomplicated appendicitis. In six patients locally or advanced purulent peritonitis was found; in two of the uncomplicated cases no peritonitis was described

### Intraoperative and histopathological findings

The intraoperative degree of inflammation of the appendix is described in Table 2b. In 1303 patients, 873 (67%) patients with uncomplicated appendicitis, 136 (10.4%) gangrenous appendicitis and 260 (20%) perforated appendicitis were observed. The remaining 34 patients were specified otherwise. Of the patients with normal inflammatory markers, 19 patients had uncomplicated appendicitis, three had gangrenous appendicitis, and one patient had perforated appendicitis.

Histopathological findings are described in Table 2c. Of the 23 patients with normal inflammatory markers, the vast majority of surgical diagnoses were confirmed by the pathologist. However, discrepancies were found in three of the four patients with complicated appendicitis. One patient had gangrenous appendicitis according to the surgeon but turned out to have uncomplicated appendicitis according to the histology report. Another case vice versa: the surgeon scored uncomplicated appendicitis, but the pathologist described gangrenous appendicitis. Lastly, one specimen was described as perforated appendicitis according to the histology report; however no perforation was seen intraoperatively (gangrenous appendicitis). As a result, four patients with normal inflammatory markers had a final diagnosis of complicated appendicitis: three patients had gangrenous appendicitis and one had perforated appendicitis.

### Subgroup of paediatric patients

A subgroup of 485 children (< 18 years of age) operated for acute appendicitis was included for secondary analysis. In 10 children both CRP and WBC were normal (Table S1). No significant differences were seen in baseline characteristics comparing among paediatric patients with normal CRP and WBC and children with elevated inflammatory markers. Within the subgroup of children with normal inflammatory parameters, no cases of complicated appendicitis were seen (Table S2).

### Subgroups of adult cohort according to gender

Young females are a special subgroup when it comes to appendicitis, as women who present with abdominal pain in the right iliac fossa have more differential diagnoses than similar presentations in men. Within this subgroup of adult young females younger than 40 years (Table S3), 10/318 (3.1%) women had appendicitis despite normal inflammatory markers, compared to 5 of 332 (1.5%) young adult men. No cases of complicated appendicitis were seen in this female group.

## Discussion

In only 1.8% of patients with histopathological confirmed appendicitis normal inflammatory markers were found. Surgical and histopathological evaluation confirmed complicated appendicitis in four of these 23 patients.

The patients with appendicitis but normal inflammatory markers had less frequent migration of pain. Other symptoms, e.g. vomiting and localized or diffuse peritonitis, were not significantly different compared to those with elevated inflammatory parameters. The duration of symptoms could affect the discriminative value of the level of inflammatory markers in predicting or excluding acute appendicitis [13, 19]. Atema et al. showed a negative predictive value of maximum 90% of normal inflammatory parameters for acute appendicitis, regardless of the duration of symptoms within a range of 5 days [4]. Therefore, they concluded that normal inflammatory markers cannot sufficiently exclude the suspected diagnosis of acute appendicitis within this 5-day range. Our findings are in line with this statement. We found that one third of the patients with normal laboratory results had symptoms for more than 48 h. This percentage was higher in the normal inflammatory parameter group compared to the increased inflammatory parameters group, although not significantly. In this cohort only duration of symptoms more or less than 48 h was noted.

In present study, the reference range of WBC was considered to be ≤ 11 × 10^9^/L. This cut-off value could be questioned. Firstly, due to inter-hospital diversity in measure equipment, no exact value is generally used. Secondly, cut-off values for children differ from values for adults. Generally, the younger the child, the higher the WBC value is considered to be within range. These cut-offs vary between centres too. To keep the analysis pragmatic and clear, we have chosen to generalize the WBC cut-off. Logically, this underestimates the number of children with normal infection parameters. In our cohort, the proportion of children with for this study considered normal inflammatory markers was 2.1% (10/485). In adults this was 1.8%.

Of all patients with histopathological confirmed acute (uncomplicated or complicated) appendicitis, 1.8% had normal inflammatory markers. Dayawansa et al. showed in a retrospective case-control study that of 281 adult patients with histologically proven appendicitis, 24 patients (8.5%) had normal WBC and CRP level at initial presentation at emergency department [17]. This rate exceeds the 1.8% in our cohort. This difference could be explained by their different cut-off values of WBC (< 12 × 10^9^/L compared to < 11 × 10^9^/L) and CRP levels (< 5 mg/L compared to ≤ 5 mg/L). Secondly, Dayawansa et al. describes that repeat bloods were taken preoperatively. These blood samples are a better representation of the final diagnosis found during operation. Only 3 patients with acute appendicitis showed repeatedly normal WBC and CRP levels. This would lead to only 1.1% (instead of 8.5%) of all patients with acute appendicitis in their cohort. In our study, we did not repetitively take blood samples. However, most of our patients (87.3%) were operated within 24 h after admission. Therefore, it could be said that the blood samples are representative for the histopathological diagnosis in our cohort.

Previous meta-analysis showed that inflammatory markers are of discriminative value in perforated appendicitis [3]. In our cohort, four patients with normal WBC and CRP levels had a final diagnosis of complicated appendicitis, of which one perforated appendicitis. It is not entirely clear why some patients with acute, and in some cases even complicated appendicitis, have normal laboratory results. One explanation could be that in some cases a viral infection can be the cause of inflammation of the appendix [20]. Furthermore, signs of infection as fever or elevated inflammatory markers are in some cases just not always present (e.g. in elderly).

## Limitations

The cohort of our initial study contains patients that underwent surgery for suspected appendicitis. Therefore, no positive or negative predictive value could be determined and no diagnostic accuracy could be analysed. Future research should include all patients presenting with abdominal pain at the emergency department in whom inflammatory parameters are determined. Secondly, while this is a multicentre study, the use of only one cut-off value could be questioned. Since there is no national guideline, we chose the range most hospitals used. A third limitation is that the duration between blood samples and appendectomy differs in our cohort. Finally, a form of information bias may be present due do the study design. The specific cases of combined complicated appendicitis and normal inflammatory parameters were retrospectively checked in each participating hospital to be sure they were no typing error in the database.

Combined normal WBC and CRP levels are seen in about 2 per 100 patients with confirmed acute appendicitis and can, although rarely, be found in patients with complicated appendicitis.

## Supplementary Information

ESM 1 (DOCX 37 kb)
