# Supplementary material for: Normal inflammatory markers and acute appendicitis: a national multicentre prospective cohort analysis
Source: Int J Colorectal Dis. 2021 Apr 27;36(7):1507–13. doi: 10.1007/s00384-021-03933-7 (PMC8195794; doi:10.1007/s00384-021-03933-7)
Supplement: Supplementary file 1 — (DOCX 37 kb) [file 384_2021_3933_MOESM1_ESM.docx]

**Supplements**

**Table S1 Baseline characteristics of patients <18 years of age with and without elevated inflammatory**

| Variable | | | Total (n=485) | Patients without elevated inflammatory markers (n=10) | Patients with elevated inflammatory markers (n=475) | p-value |
| --- | --- | --- | --- | --- | --- | --- |
| Age, median (IQR) in years  *Missing n=0* | | | 12 (9-15) | 10.5 (9-11) | 12 (9-15) | 0.06 |
| Sex, male (%)  *Missing n=0* | | | 297 (61.2) | 8 (80) | 289 (60.8) | 0.33* |
| ASA (%)  *Missing n=0* | I-II | | 484 (99.8) | 10 (100) | 474 (99.8) | 1.00* |
|  | III-IV | | 1 (0.2) | 0 (0) | 1 (0.2) |  |
| Duration of symptoms <2 or >2 days (%)  *Missing n=10* | | <2 days | 373 (76.9) | 6 (60.0) | 367 (77.3) | 0.23* |
|  |  | >2 days | 102 (21.0) | 4 (40.0) | 98 (20.6) |  |
| Migration of pain (%)  *Missing n=13* | | | 196 (40.4) | 3 (30) | 193 (40.6) | 0.74* |
| Nausea (%)  *Missing n=13* | | | 358 (73.8) | 8 (80) | 350 (73.7) | 1.00* |
| Vomiting (%)  *Missing n=9* | | | 272 (56.1) | 5 (50) | 267 (56.2) | 0.75* |
| Fever, ≥38.5 ̊ Celsius (%)  *Missing n=28* | | | 124 (25.6) | 0 (0) | 124 (26.1) | 0.12* |
| Peritonitis (%)  *Missing n=21* | | Localized | 414 (85.4) | 10 (100) | 404 (85.1) | 0.61* |
|  |  | Diffuse | 50 (10.3) | 0 (0) | 50 (10.5) |  |
| Inflammatory  markers  *Missing n=0* | WBC, mean (SD) 10˄9/L | | 15.1 (5.0) | 8.9 (2.1) | 15.2 (5.0) | >0.001 |
|  | CRP, median (IQR) mg/L | | 28 (11-81) | 3 (1-4) | 30 (12-83) | >0.001 |
| Time to surgery, median (IQR) in hours  *Missing n=22* | | | 5.2 (3.5-10.4) | 5.9 (4.0-8.9) | 5.2 (3.5-10.4) | 0.82 |
| Type of appendectomy ITT (%)  *Missing n=3* | Laparoscopic | | 288 (59.4) | 5 (50) | 283 (59.6) | 1.00* |
|  | Open | | 194 (40.0) | 4 (40) | 190 (40.0) |  |
| Duration of surgery, median (IQR) in min  *Missing n=55* | | | 36 (28-47) | 40 (20-58) | 36 (29-46) | 0.80 |
| LOS, median (IQR) in days  *Missing n=0* | | | 2 (1-3) | 2 (1.8-2.3) | 2 (1-3) | 0.83 |
| Abbreviations: ASA, American Society of Anesthesiologists; IQR, inter quartile range; ITT, intention to treat; LOS, length of stay.  * Fisher’s exact test was used | | | | | | |

**Table S2. Imaging results and intraoperative and histopathological findings of patients <18 years of age**

| Variable | Total (n=485) | Patients without elevated inflammatory markers (n=10) | Patients with elevated inflammatory markers (n=475) |
| --- | --- | --- | --- |
| 1. Imaging results |  | | |
| Inconclusive (%) | 45 (9.3) | - | 45 (9.5) |
| Uncomplicated appendicitis (%) | 365 (75.3) | 8 (80) | 357 (75.2) |
| Perforated appendicitis (%) | 48 (9.9) | 1 (10) | 47 (9.9) |
| Inflammatory mass (%) | 15 (3.1) | - | 15 (3.2) |
| Inflammatory mass with abscess (%)* | 8 (1.6) | 1 (10) | 7 (1.5) |
| No imaging (%) | 4 (0.8) | - | 4 (0.8) |
| 1. Intraoperative findings |  | | |
| Normal appendix (%) | 2 (0.4) | - | 2 (0.4) |
| Uncomplicated appendicitis (%) | 345 (71.1) | 9 (90) | 336 (70.7) |
| Gangrenous appendicitis (%) | 41 (8.5) | - | 41 (8.6) |
| Perforated appendicitis (%) | 87 (17.9) | - | 87 (18.3) |
| Iatrogenic perforation (%) | 9 (1.9) | - | 9 (1.9) |
| Other* | 1 (0.2) | 1 (10) | - |
| 1. Histopathological findings |  | | |
| Uncomplicated appendicitis (%) | 400 (82.5) | 10 (100) | 390 (82.1) |
| Gangrenous appendicitis (%) | 42 (8.7) | - | 42 (8.8) |
| Perforated appendicitis (%) | 43 (8.9) | - | 43 (9.1) |
| *Intraoperatively, one specimen was scored as ‘tumor of the appendix’, but turned out to be gangrenous appendicitis at histological examination. | | | |

**Table S3. Imaging results and intraoperative and histopathological findings of female adult patients <40 years of age**

| Variable | Total (n=328) | Patients without elevated inflammatory markers (n=10) | Patients with elevated inflammatory markers (n=318) |
| --- | --- | --- | --- |
| 1. Imaging results |  | | |
| Inconclusive (%) | 21 (6.4) | - | 21 (6.6) |
| Uncomplicated appendicitis (%) | 270 (82.3) | 10 (100) | 260 (81.8) |
| Perforated appendicitis (%) | 27 (8.2) | - | 27 (8.5) |
| Inflammatory mass (%) | 8 (2.4) | - | 8 (2.5) |
| Inflammatory mass with abscess (%)* | 2 (0.6) | - | 2 (0.6) |
| No imaging (%) | - | - | - |
| 1. Intraoperative findings |  | | |
| Normal appendix (%) | 4 (1.2) | - | 4 (1.3) |
| Uncomplicated appendicitis (%) | 264 (80.5) | 10 (100) | 254 (79.9) |
| Gangrenous appendicitis (%) | 19 (5.8) | - | 19 (6.0) |
| Perforated appendicitis (%) | 37 (11.3) | - | 37 (11.6) |
| Iatrogenic perforation (%) | 4 (1.2) | - | 4 (1.3) |
| 1. Histopathological findings |  | | |
| Uncomplicated appendicitis (%) | 280 (85.4) | 10 (100) | 270 (84.9) |
| Gangrenous appendicitis (%) | 25 (7.6) | - | 25 (7.9) |
| Perforated appendicitis (%) | 23 (7.0) | - | 23 (7.2) |
| *Intraoperatively, one specimen was scored as ‘tumour of the appendix’, but turned out to be gangrenous appendicitis at histological examination. | | | |
